# Risk of Remote Seizures After Perinatal Ischemic Stroke

**DOI:** 10.15844/pedneurbriefs-30-8-1

**Published:** 2016-08

**Authors:** David G. Ritacco

**Affiliations:** 1 Division of Neurology, Ann & Robert H. Lurie Children’s Hospital of Chicago, Chicago, IL; 2 Department of Pediatrics, Northwestern University Feinberg School of Medicine, Chicago, IL

**Keywords:** Epilepsy, Brain ischemia, Stroke

## Abstract

Investigators from the University of California San Francisco and Kaiser Permanente Medical Centers in Oakland report outcome of perinatal arterial ischemic stroke (PAIS) in a population-based cohort, with particular attention to the incidence of seizures.

Investigators from the University of California San Francisco and Kaiser Permanente Medical Centers in Oakland report outcome of perinatal arterial ischemic stroke (PAIS) in a population-based cohort, with particular attention to the incidence of seizures. The authors took their stroke population from those in the Kaiser Permanente Stroke Study, drawn from 2.5 million patients <18 years old followed through a single integrated Northern California health system between 1993 and 2007, with median age at follow-up of 7.1 years. This cohort is thought to well represent the regional population as a whole, under-representing only the socioeconomic extremes. The selected population was assembled by retrospective chart screening with a multilayered process (including searching diagnostic codes, keywords, radiology reports), requiring CT or MR imaging findings and matching clinical features. They found 48 children diagnosed with stroke in infancy and 39 who came to diagnosis later (PPIS). Forty of those diagnosed in infancy presented with perinatal seizures. They found subsequent unprovoked seizures in 37 children, or 42.5%, adjusted to 54% cumulative incidence at 10 years, as well as a heavy burden of active epilepsy, defined as at least one unprovoked seizure as well as ongoing anticonvulsant treatment at last contact, in 24 children, or 27.5%, 40% cumulative at 10 years. Both were worse in those who presented with neonatal seizures: 69% cumulative incidence of remote seizures and 54% of active epilepsy at 10 years, respectively. The authors noted that the annual incidence of remote seizure was greatest in the first year at 20%, rising to 46% at 5 years, before starting to level off at about 10 years. Those with stroke diagnosis in the neonatal period were more likely to have multiple infarcts (29% vs 3 %), but less likely to have a CP diagnosis (50% vs 85%) and achieved walking at a younger age 13 vs 16 months). [1]

COMMENTARY. This interesting study includes those in the less commonly described group of PPIS as well as those with PAIS. The incidence of arterial stroke presenting in the newborn period is about 1 in 4000 [2], and an earlier report drawn from this cohort suggests about the same frequency for the number of births included [3]. This study finds a high rate of epilepsy compared to prior reports, but the published incidence of seizures and epilepsy after perinatal ischemic stroke varies widely, with those for PAIS between 16 and 56% [4] and those for PPIS 38-42% [5, 6], affected at least in part by differing periods of follow-up. It should be noted that the reported cumulative incidence is calculated from the time to first remote seizure, allowing estimation of seizure occurrence by a given age (a goal of the authors, and useful for counseling parents at the time of diagnosis).

The authors admit that although those with PAIS and PPIS have the same illness, recruitment into these groups is subject to ascertainment bias. This may account for the more severe cerebral palsy seen in the PPIS group, the majority of which came to diagnosis because of motor findings (34/39 = 87%, compared to 8/48 =17% of the PAIS group), and may contribute to the more severe seizure outcome in the PAIS group, the majority of which came to diagnosis because of seizures (40/48 = 83%, compared to 5/39 =13% of the PAIS group).
